# Amino-3,5-Dicyanopyridines Targeting the Adenosine Receptors. Ranging from Pan Ligands to Combined A1/A2B Partial Agonists

**DOI:** 10.3390/ph12040159

**Published:** 2019-10-22

**Authors:** Daniela Catarzi, Flavia Varano, Katia Varani, Fabrizio Vincenzi, Silvia Pasquini, Diego Dal Ben, Rosaria Volpini, Vittoria Colotta

**Affiliations:** 1 Dipartimento di Neuroscienze, Psicologia, Area del Farmaco e Salute del Bambino, Sezione di Farmaceutica e Nutraceutica, Università degli Studi di Firenze, Via Ugo Schiff, 6, 50019 Sesto Fiorentino, Italy; flavia.varano@unifi.it (F.V.); vittoria.colotta@unifi.it (V.C.); 2 Dipartimento di Scienze Mediche, Sezione di Farmacologia, Università degli Studi di Ferrara, Via Fossato di Mortara 17-19, 44121 Ferrara, Italy; vrk@unife.it (K.V.); fabrizio.vincenzi@unife.it (F.V.); silvia.pasquini@unife.it (S.P.); 3 Scuola di Scienze del Farmaco e dei Prodotti della Salute, Università degli Studi di Camerino, via S.Agostino 1, 62032 Camerino (MC); Italy; diego.dalben@unicam.it (D.D.B.); rosaria.volpini@unicam.it (R.V.)

**Keywords:** G protein-coupled receptors, adenosine A_2B_ receptor ligands, adenosine A_1_ receptor ligands, aminopyridine-3,5-dicarbonitriles, ligand-adenosine receptor modeling studies

## Abstract

The amino-3,5-dicyanopyridine derivatives belong to an intriguing series of adenosine receptor (AR) ligands that has been developed by both academic researchers and industry. Indeed, the studies carried out to date underline the versatility of the dicyanopyridine scaffold to obtain AR ligands with not only a wide range of affinities but also with diverse degrees of efficacies at the different ARs. These observations prompted us to investigate on the structure–activity relationships (SARs) of this series leading to important previously reported results. The present SAR study has helped to confirm the 1*H*-imidazol-2-yl group at R^2^ position as an important feature for producing potent AR agonists. Moreover, the nature of the R^1^ substituent highly affects not only affinity/activity at the hA_1_ and hA_2B_ ARs but also selectivity versus the other subtypes. Potent hA_1_ and hA_2B_ AR ligands were developed, and among them, the 2-amino-6-[(1*H*-imidazol-2-ylmethyl)sulfanyl]-4-[4-(prop-2-en-1-yloxy)phenyl]pyridine-3,5-dicarbonitrile (**3**) is active in the low nanomolar range at these subtypes and shows a good trend of selectivity versus both the hA_2A_ and hA_3_ ARs. This combined hA_1/_hA_2B_ partial agonist activity leads to a synergistic effect on glucose homeostasis and could potentially be beneficial in treating diabetes and related complications.

## 1. Introduction

Adenosine is a ubiquitous autacoid that has been reported to play important roles in various tissues by acting through four different adenosine receptors (ARs) classified as A_1_, A_2A_, A_2B_, and A_3_ receptors [1]. These ARs belong to the G protein–coupled receptors superfamily, and each of them has different functions although with some overlap. Depending on their coupling to G-protein, they can either increase or decrease intracellular cAMP levels by affecting adenylate cyclase (AC) activity. However, other effector systems have been proposed [2]. Adenosine has the highest affinity for the A_1_ and A_2A_ ARs, the lowest for A_2B_AR, and intermediate affinity for the A_3_ subtype. Selective agonists and antagonists have been developed for each of the ARs [3,4], and their discovery opened up new avenues for potential drugs treatment of a variety of conditions such as neurodegenerative disorders, chronic inflammatory diseases, asthma, and many other pathophysiological states that are known to be associated with changes in adenosine levels [3].

Dysregulated intracellular nucleotide concentrations can be produced by altered and defective cellular metabolism leading to important metabolic pathologies such as diabetes mellitus [3,5,6]. This disease is increasing worldwide due to aging, incorrect lifestyle, and eating habits, and its complications are of great clinical and socioeconomic impact. All four ARs are involved in the regulation of islet hormone secretion and have the potential of being drug targets for the treatment of diabetes [3]. Currently, much attention has been devoted to A_2B_AR agonists as potential new drugs for diabetes treatment, even if there is discrepancy in the studies reported in literature, which may be ascribed to the different experimental conditions employed [3,7]. However, it is reported that agonists of this AR subtype may be useful for the therapy of this pathology [3]. 

**Figure 1:**
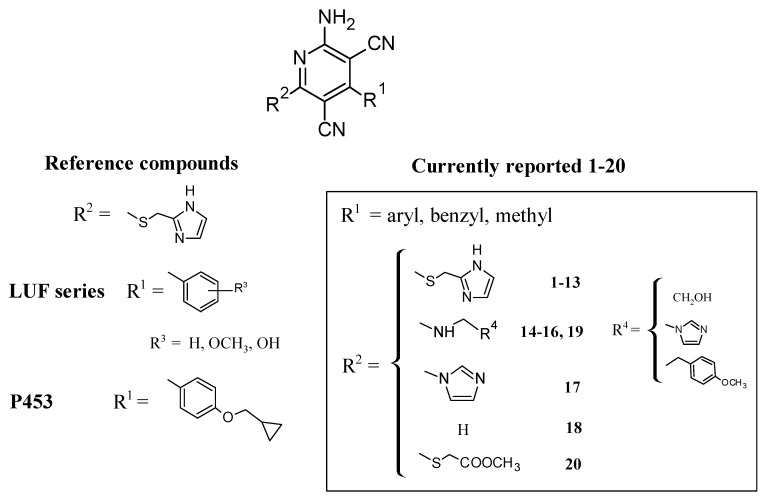
Lead structures and currently reported amino-3,5-dicyanopyridine-based adenosine receptor ligands.

The role of the A_1_AR in diabetes seems to be clearer [3,8]. In fact, improvement of insulin sensitivity and glucose homeostasis by A_1_ activation has been well recognized for many years, thus the promotion of A_1_AR agonists as drug candidates [5,6]. Since full A_1_AR agonists are generally characterized by cardiovascular side effects, partial A_1_AR agonists could represent the best choice to avoid cardiovascular complications [9]. For this reason, a synergistic effect on glucose homeostasis targeting both A_1_ and A_2B_ ARs could be of interest for treating diabetes and its complications. It is also worth noting that A_1_AR is involved in lipid metabolism, so the development of A_1_AR agonists as lipolytic agents is reported to be useful in cardiovascular disease–associated pathologies [10]. While the pathophysiological roles of the A_1_AR are well known, the A_2B_ subtype is the least characterized because of the paucity of potent and selective agonists. BAY-606583 (2-{[6-Amino-3,5-dicyano-4-(4-(cyclopropylmethoxy)phenyl)pyridin-2-yl]thio}acetamide) is one of the most potent and selective A_2B_AR agonists known to date [11]. However, we recently produced a new series of amino-3,5-dicyanopyridines as BAY-606583 analogues [12]; most of the compounds are potent human (h) A_2B_ AR partial agonists. Among them, the 2-{[(1*H*-imidazol-2-yl)methyl]thio}-6-amino-4-[4-(cyclopropylmethoxy)phenyl]pyridine-3,5-dicarbonitrile (P453, Figure 1) turns out to be three-fold more active than BAY-606583, although it is less selective. Compound P453 was used for experiments in in vitro models of short-term synaptic plasticity and ischemic-like condition [13]. In particular, compound P453 was shown to be a functional A_2B_AR agonist whose activation decreases PPF (paired pulse facilitation) by increasing glutamate release at presynaptic terminals and delays A_1_AR-mediated fEPSP inhibition during a two-minute oxygen and glucose deprivation (OGD) insult in the rat CA1 hippocampus. 

From a structure–activity point of view, the new findings confirm the 1*H*-imidazol-2-yl group in the R^2^ pendant as an important feature for producing potent AR agonists. Certain 2-amino-4-aryl-6-(1*H*-imidazol-2-yl-methylsulfanyl)-pyridine-3,5-dicarbonitrile compounds, belonging to the LUF series (Figure 1), were already reported to display nanomolar affinity for all the ARs, including the A_2B_ subtype for which they showed, in general, considerable affinity and efficacy [14]. The growing interest in this class is also dictated by the fact that non-nucleoside AR agonists seem to be more versatile for pharmacological studies, showing fewer species differences than the adenosine-like ones [4]. Moreover, the studies carried out to date are sufficient to underline the versatility of the amino-3,5-dicyanopyridine scaffold for producing AR ligands with not only a wide range of affinities but, interestingly, with different degrees of efficacy at the different ARs [11,12,14,15,16,17,18,19].

On this basis, the 1*H*-imidazol-2-yl group was used to design other amino-3,5-dicyanopyridine derivatives in order to deeply investigate the influence of the R^1^ substituent at the 4-position on AR affinity and selectivity. Moreover, other modifications were performed at R^2^ to enrich what is known about how the nature of the substituent at this position modulates AR affinity and selectivity.

## 2. Results

### 2.1. Chemistry

**Scheme 1:**
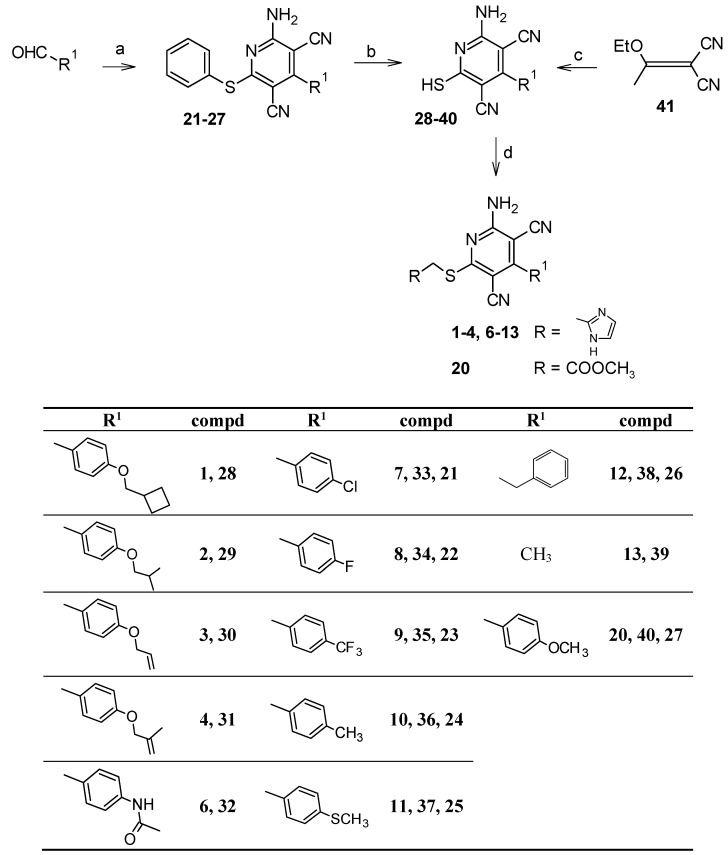
**Reagents and conditions.** (a) malononitrile, thiophenol, basic Al_2_O_3_, H_2_O, 100 °C; (b) Na_2_S, anhydrous DMF, 80 °C; 1N HCl, room temperature (rt); (c) 2-cyanothioacetamide, EtONa, absolute EtOH, reflux; d) RCH_2_Cl, NaHCO_3_, anhydrous DMF, rt.

**Scheme 2:**
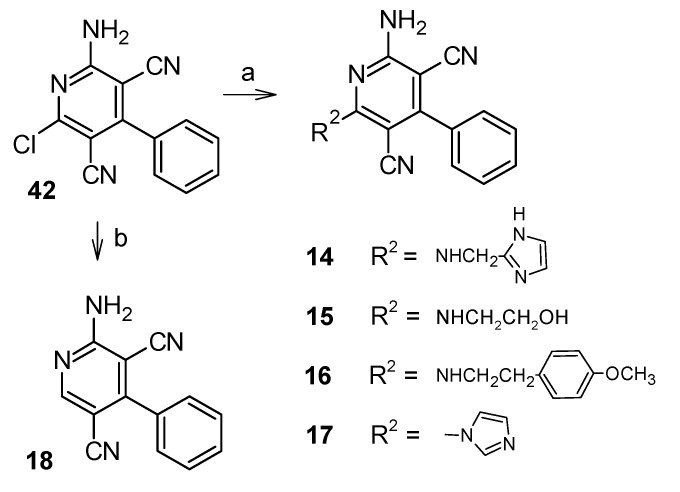
**Reagents and conditions.** (a) 1-(1H-imidazol-2-yl)methanamine for **14**, ethanolamine for **15**, 2-(4-methoxyphenyl)ethanamine for **16**, or imidazole (for **17**), triethylamine, THF/EtOH 2:1, reflux; (b) H_2_, 10% Pd/C, absolute EtOH, 30 Psi.

**Scheme 3:**
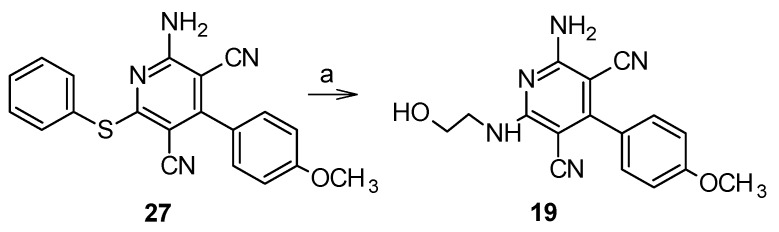
Reagents and conditions. (a) 2-aminoethanol, DMF, 100 °C.

Compounds **1**–**4**, **6**–**20**, and their corresponding intermediates were synthesized according to the procedure reported in Scheme 1, Scheme 2 and Scheme 3. Following the same procedures, the 2-(((1*H*-Imidazol-2-yl)methyl)thio)-6-amino-4-(4-ethoxyphenyl)pyridine-3,5-dicarbonitrile **5**, previously reported in reference [12], was prepared. For the synthesis of derivatives **1**–**4**, **6**–**13**, and **20**, the strategic intermediates are represented by the 6-sulfanyl-substituted compounds **28**–**40** [12,14,18,20,21,22,23], which were prepared as depicted in Scheme 1. The suitable aldehyde was reacted with malononitrile and thiophenol, in boiling water, in the presence of a catalytic amount of basic alumina to yield the 4-aryl- and 4-benzyl-6-phenylsulfanyl-derivatives **21**–**27** [18,24,25,26], with moderate to good yield. When these latter were treated with anhydrous sodium sulfide in anhydrous DMF at 80 °C, followed by acidification with HCl, the free thiol **28**–**38** [12,18,20,21], **40** [14] were obtained in high yields. The preparation of the 4-methyl-6-sulfanyl compound **39** [22,23] was performed by reacting the (1-ethoxyethylidene)propanedinitrile **41** [22] with 2-cyanothioacetamide in boiling ethanol containing EtONa. The target dicyanopyridines **1**–**4**, **6**–**13**, and **20** were obtained by reaction of the 6-sulfanyl intermediates **28–40** with 2-(bromomethyl)-1*H*-imidazole hydrobromide (**1**–**4**, **6**–**13**) or methyl chloroacetate (**20**) in the presence of sodium hydrogen carbonate at room temperature (rt). 

To replace the thiomethyl linker at the 6-position with an aminomethyl bridge, compounds **14**–**16** were synthesized (Scheme 2) starting from the 6-chloro derivative **42**, obtained with a reported procedure [27], and the suitable commercially available amine. The reaction was carried out in the presence of triethylamine and in a refluxing mixture of THF/EtOH. The same procedure was applied for the synthesis of the 6-(1*H*-imidazol-1-yl)-derivative **17**, which was obtained in high yield. Elimination of the 6-substituent was realized in compound **18** which was synthesized by catalytic reduction (10% Pd/C) of **42** with hydrogen at 30 Psi in absolute EtOH.

Finally, the 6-[(2-hydroxyethyl)amino]-derivative **19** was synthesized (Scheme 3) starting from the intermediate **27** [24] by exploiting the good property of the 6-phenylthio function as the leaving group in nucleophilic substitution [15]. The reaction was performed at 100 °C in DMF using an excess of 2-aminoethanol as nucleophile.

### 2.2. Pharmacological Assays 

The amino-3,5-dicyanopyridines **1**–**20** were tested for their affinity at hA_1_, hA_2A_, and hA_3_ ARs, stably transfected in Chinese Hamster Ovary (CHO) cells, and were also studied as hA_2B_ agonists by evaluating their stimulatory effect on cAMP production in CHO cells, stably expressing the hA_2B_ AR. Compounds **3**, **8**, and **11**, the most interesting in terms of combined affinity/activity at hA_1_ and hA_2B_ ARs, were evaluated for their A_1_ pharmacological profile in the cAMP assay, where each compound was tested to assess its capability to modulate Forskolin-stimulated cAMP levels in the absence or presence of the A_1_AR agonist 2-chloro-*N*^6^-cyclopentyladenosine (CCPA) (1 nM). All pharmacological data are reported in Table 1 and Table 2. 

### 2.3. Molecular Docking Studies 

To simulate the binding mode of the herein reported dicyanopyridines at hA_1_ and hA_2B_ ARs, molecular docking studies were performed on the cryo-EM structure of the agonist-bound hA_1_AR (pdb code: 6D9H; 3.6-Å resolution [28]) and on a homology model of the hA_2B_AR developed by using the crystal structure of agonist-bound hA_2A_AR as a template (pdb code: 2YDO; 3.0-Å resolution [29]). The obtained hA_2B_AR homology model was checked using the Protein Geometry Monitor application within MOE (Molecular Operating Environment 2019.0101) [30] by inspecting the structural quality of the protein model (backbone bond lengths, angles, and dihedrals, Ramachandran φ-ψ dihedral plots, and quality of side chain rotamer and non-bonded contact). The two AR structures were then used as target for the docking analysis of the synthesized derivatives. The docking studies were performed using the MOE docking tool (induced fit docking and optimization protocol) and Gold software [31]. For each compound, the top-score docking pose at each AR model, according to at least two out of three scoring functions, was selected for final ligand-target interaction analysis.

**Figure 2:**
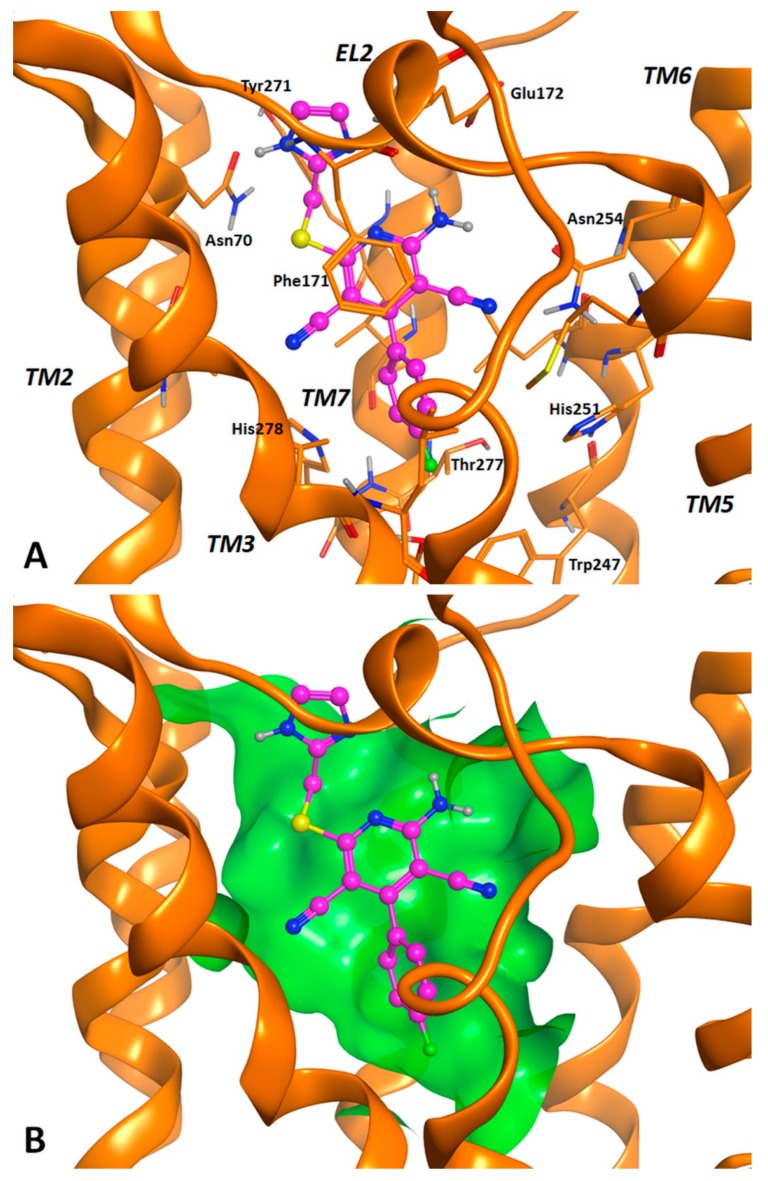
Docking conformations at hA_1_AR. The simulated binding mode of compound **8** is shown (**A**) with detail of key receptor residues involved in ligand-target interaction or (**B**) with representation of the binding cavity as molecular surface.

**Figure 3:**
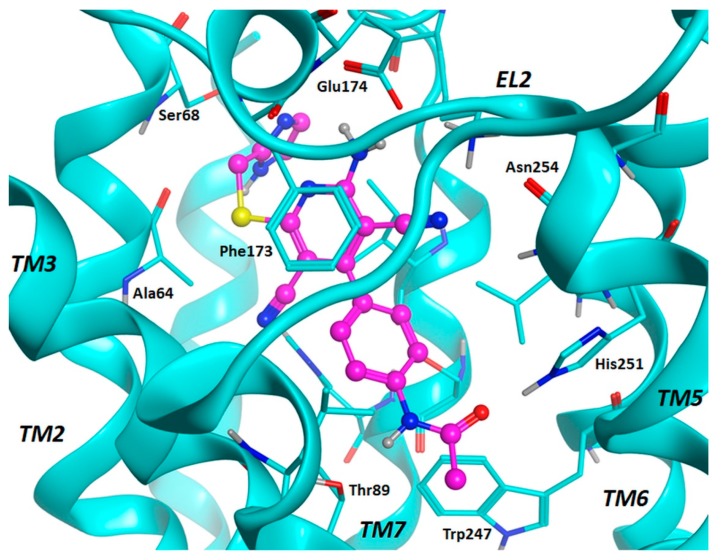
Docking conformations at hA_2B_AR. The simulated binding mode of compound **6** is shown, with detail of key receptor residues involved in ligand–target interaction.

Docking conformations at hA_1_ and hA_2A_ ARs of selected dicyanopyridine compounds are reported in Figure 2 and Figure 3, respectively (see the Discussion Section).

## 3. Discussion

### 3.1. Structure–Activity Relationships

The biological and pharmacological data for the newly synthesized amino-3,5-dicyanopyridine derivatives **1**–**20** are reported in Table 1 and Table 2 together with those of the reference compounds LUF5833 (2-amino-6-[(1*H*-imidazol-2-ylmethyl)sulfanyl]-4-phenylpyridine-3,5-dicarbonitrile) [14] and P453 [12]. All the derivatives that interact with the hA_2B_AR (**2, 3, 5**–**8, 10**–**12**) display EC_50_ values from 12 to 260 nM behaving as partial agonists, with the only exception being compound **6**, previously reported as trifluoroacetate salt [32], which shows a full agonist profile. Some compounds (**17**–**20**), selected among those that had no activity at the hA_2B_AR were also evaluated to investigate their antagonistic effect on cAMP production stimulated by 5′-(*N*-ethylcarboxamido)adenosine (NECA) 100 nM in hA_2B_CHO cells. These functional studies reveal their inability to inhibit NECA-stimulated cAMP levels (I% ≤ 3) (data not shown). Most of the compounds have generally modest to null hA_2A_ and hA_3_ AR affinity with the only exception of the two pairs of compounds **6** and **10** and **5** and **6** that bind, respectively, the hA_2A_ and hA_3_ ARs with nanomolar affinity. Best results have been obtained in the hA_1_AR binding experiments. In particular, all the 6-(imidazole-2-ylmethyl)sulfanyl derivatives reported herein (**1**–**13**), with the only exception being compounds **1**, **2**, **9**, and **12**, have from high to good hA_1_AR affinity. In particular, some compounds showed *K*_i_ values in the low nanomolar range and in general below 10 nM. Functional studies at the hA_1_ subtype (data are reported in Table 2) indicated that the tested compounds **3**, **8**, and **11** behave as partial agonists and that their relative potencies correlate well with the *K*_i_ values obtained in the hA_1_ binding experiments. All the newly synthesized compounds showed different and not easily predictable behavior depending on both R^1^ and R^2^ substituents. However, the data shown in Table 1 indicate that the binding at the hA_2B_AR subtype depends strictly on the substitution at the R^1^ position [12]. 

Modifications of the *para*-substituent at the R^1^ pendant of the lead compound P453 were performed to evaluate the influence on hA_2B_AR activity and selectivity. 6-(Imidazole-2-ylmethyl)sulfanyl derivatives **1**–**5,** bearing different cycloalkyl- or cycloalkenyl-methyloxy substituents at R^1^ position, were synthesized. Only compounds **3** and **5** maintain the hA_2B_AR activity in the nanomolar range, while derivative **2** and derivatives **1** and **4** are, respectively, scarcely active or inactive, probably due to the different steric hindrance of the *para*-substituent with respect to P453. In particular, derivative **5** is endowed with hA_2B_AR activity comparable to that of the lead P453, despite a low or null selectivity versus the other AR subtypes. Introduction of a *para*-acetamido substituent on the 4-phenyl moiety led to compound **6**, which is the only amino-3,5-dicyanopyridine in the whole series having a full agonist profile at the hA_2B_AR. The *para*-acetamido substituent, in addition to possessing an oxygen atom as H-bond acceptor, also contains the NH donor group that could have some influence in determining the pharmacological profile of this derivative as observed for the dicyanopyridine of the LUF series (Figure 1) [33,34]. 

Activity at the hA_2B_AR is also affected by the electronic effects of the *para*-substituent on the 4-phenyl ring. In fact, the corresponding EC_50_ values fall in the low nanomolar range for compounds **8** (*para*-fluoro) and **11** (*para*-thiomethyl) and move to higher values by passing to derivatives **10** (*para*-methyl) and **7** (*para*-chloro), up to the total inactivity of **9**, bearing a *para*-trifuoromethyl group. Homologation of the 4-phenyl moiety of LUF5833 led to the 4-benzyl-susbstituted derivative **12**, which maintains modest hA_2B_ activity. In contrast, its replacement with a methyl group was highly detrimental leading to compound **13**, which is completely avoid of any activity at the hA_2B_AR subtype. In this first set of compounds (**1**–**13**), bearing a 6-(imidazole-2-ylmethyl)sulfanyl moiety on the dicyanopyridine scaffold, the hA_1_AR affinity depends on the *para*-phenyl substitution as observed for hA_2B_ receptor activity trend. In fact, many compounds show mixed hA_1_/hA_2B_ combined affinity/activity. Looking at Table 1, we can observe different activity profiles—for example, compound **6**, whose biological data fall in the same range of values (EC_50_ at hA_2B_AR and *K*_i_ at the other subtypes) can be considered in effect as a pan ligand. Compound **3** is interesting in terms of its combined hA_1_/hA_2B_ AR partial agonist activity, exhibiting a good trend of selectivity versus both the hA_2A_ and hA_3_ subtypes. The data of derivatives **8** and **11,** which lose a little in selectivity but behave as hA_1_/hA_2B_ partial agonists, are also noteworthy. Moreover, compound **8,** binding the hA_1_AR in the sub-nanomolar range, is the most potent hA_1_AR ligand among the herein reported dicyanopyridine series. When the 6-sulfanyl linker of LUF5833 was replaced by a 6-amino bridge, compound **14** was obtained. A dramatic decrease of hA_2B_AR activity was observed with respect to the LUF derivative but the effect was detrimental also for the other ARs. Replacement of the imidazolyl moiety at R^2^ position with different substituents in terms of both steric hindrance and capability to engage hydrogen-bonding led to derivatives **15**–**20**, which show null affinity/activity for each of the AR subtypes. The only exception are compounds **15** and **20** endowed with hA_1_AR binding affinity in the micromolar range. Compounds **17**–**20**, besides being inactive as hA_2B_ agonists, do not even work as antagonists.

### 3.2. Molecular Docking Investigation and In Silico ADMET Prediction.

The simulated binding mode at both the hA_1_ and hA_2B_ AR structures, generally associated to the best score, presents the herein reported compounds oriented similarly to analogous derivatives previously reported as hAR ligands (Figure 2A) [12,35,36]. In detail, the pyridine scaffold is located in the AR cavity in correspondence to the purine moiety of Ado observed at the hA_1_AR cryo-EM structure and at the hA_2A_AR template, and interacts with the side chains of hA_1_AR residues Phe171 extracellular loop (EL) 2 and Ile274^7.39^ (Phe173 and Ile276^7.39^ in hA_2B_AR). For clarity, in this section, derivative **8** has been considered the reference compound to define the position of the substituents on the pyridine nucleus. Thus, starting from the N1 position, the amino and the sulfanyl functions occupy positions 2 and 6, respectively. The 3-cyano and 2-amino groups make H-bonds with the amide function of Asn254^6.55^. An additional H-bond is observed between the 2-amino group and the hA_1_AR residue Glu172 (EL2, Glu174 in hA_2B_AR), while the 5-cyano group points toward transmembrane (TM) 2 (i.e., Ala66^2.61^ in hA_1_AR, Ala64^2.61^ in hA_2B_AR) and TM7 (i.e., His278^7.43^ in hA_1_AR, His280^7.43^ in hA_2B_AR) residues. The 2-sulfanyl substituent is located at the entrance of the binding cavity, providing interaction with residues of TM1, TM2, and TM7. Binding data show that in general an aryl moiety at R^1^ is the type of substituent providing highest affinity at ARs. Its replacement with a smaller methyl group (compound **13**) or a larger (and differently oriented) benzyl substituent (**12**) leads to a significant decrease (if not loss) of affinity. In this sense, the 4-aryl moiety well fits the depth of the binding cavity at both receptors, by occupying the available space in a more complete and efficient way with respect to the methyl (**13**) or benzyl group (**12**) (Figure 2B). Looking at the *para*-position of the 4-aryl moiety, biological data show that the size of the substituent at this level is critical for compound affinity, with the highest obtained with a small group. At the hA_1_AR receptor, the space available around the *para*-position is limited, allowing the insertion of small residues and not well tolerating ramified substituents. Accordingly, compounds bearing a bulky, non-linear *para*-substituent (compounds **4** and **6**) are endowed with lower hA_1_AR affinity with respect to other compounds of the series active in the low nanomolar range.

At the hA_2B_AR, the impact of the size of the *para*-substituent on compound affinity appears analogous to that observed at the hA_1_AR, with the exception of compound **6** bearing a *para*-acetamido group. This compound is among the most potent derivatives of the series at the hA_2B_AR. Figure 3 shows the binding mode of compound **6** at the hA_2B_AR, with indication of the key residues involved in ligand interaction. The *para*-acetamido function engages a double H-bond interaction with Thr89^3.36^ and His251^6.52^. This double polar interaction with the receptor is also given by the 5′-amide groups of the hA_2A_AR agonists NECA [29], UK-432097 (6-[2,2-di(phenyl)ethylamino]-9-[(2R,3R,4S,5S)-5-(ethylcarbamoyl)-3,4-dihydroxyoxolan-2-yl]-*N*-[2-[(1-pyridin-2-ylpiperidin-4-yl)carbamoylamino]ethyl]purine-2-carboxamide) [37] and CGS-21680 (4-[2-[[6-amino-9-(*N*-ethyl-β-D-ribofuranuronamidosyl)-9H-purin-2-yl]amino]ethyl]benzenepropanoic acid) [38], as observed from X-ray structures of the hA_2A_AR in complex with these two compounds. 

The insertion of an amide group at the 5′-position of nucleosides is a typical modification of the Ado scaffold to improve its affinity and potency at the hA_2A_ and hA_2B_ ARs. This observation helps to explain the high activity of compound **6** at the hA_2B_AR. 

The potential use of derivatives belonging to this series as therapeutic agents depends not only on their pharmacodynamics but also pharmacokinetics, the latter including Adsorption, Distribution, Metabolism, Excretion, and Toxicity (ADME/T). A preliminary study of the theoretical ADMET profiles of compound **3** was performed using the methodology developed by pKCSM, a novel approach to the prediction of pharmacokinetic and toxicity properties [39]. A complete summary of all the parameters evaluated for the selected compound is reported in the Supplementary Materials. It is worth noting that no violation of the Lipinski’s rule of five (RO5) (molecular weight (MW) < 500; log P < 5; number of H.-bond donors (HBD) < 5, and acceptors (HBA) < 10) was evidenced, making derivative **3** a promising drug-like candidate [40]. Nevertheless, while some bioavailability and toxicity parameters fall in the safety range, others indicate some points of concerns for compound **3**. In interpreting the contrasting results, it is important to consider that in silico evaluation of the ADMET parameters for a group of approved antidiabetic drugs [41] evidenced not only violation of some of the RO5 (25%) but also the toxicity parameters as the major failures (2–33%). 

## 4. Conclusions

This study, which has led to some amino-3,5-dicyanopyridines differently substituted at R^1^ and R^2^ positions, provides greater insight into the SARs of this series as AR ligands. In particular, it has emerged that the affinity/activity at the hA_1_ and hA_2B_ ARs depends strictly on the R^1^ substitution which also affects the selectivity versus the other subtypes. Moreover, the 1*H*-imidazol-2-yl group at R^2^ position was confirmed to be an essential feature for potent AR agonists. In fact, looking at the biological and pharmacological data of this series, it is usual to find a combined AR affinity/activity, even if most of the compounds have generally modest to null hA_2A_ and hA_3_ AR affinity. Many of the dicyanopyridines herein reported interact with the hA_2B_ subtype with IC_50_ values from 12 to 260 nM behaving as partial agonists. The best results were obtained in the hA_1_AR binding assays where most of the compounds show from high to good affinity at this receptor, the 2-amino-4-(4-fluorophenyl)-6-[(1*H*-imidazol-2-ylmethyl)sulfanyl]pyridine-3,5-dicarbonitrile (**8**) emerging as the most active compound with a partial agonist profile. The compounds showing a combined hA_1_/hA_2B_ AR partial agonist activity, as derivatives **3**, **8**, and **11**, deserve the greatest attention. In particular, the 2-amino-6-[(1*H*-imidazol-2-ylmethyl)sulfanyl]-4-[4-(prop-2-en-1-yloxy)phenyl]pyridine-3,5-dicarbonitrile (**3**) is active in the low nanomolar range at both these subtypes and shows a good trend of selectivity versus both the hA_2A_ and hA_3_ ARs. This combined hA_1/_hA_2B_ partial agonist activity, leading to a synergistic effect on glucose homeostasis, could be of interest in the development of potentially useful agents for treating diabetes and its complications. The in silico calculated ADMET profiles indicated that some bioavailability and toxicity parameters fall in the safety range, while others suggest some points of concerns for compound **3**. Thus, further studies will be devoted in the future to thoroughly investigate on the toxicity of compound **3**. We are however confident in considering **3** as a promising lead for the development of new antidiabetics with improved pharmacokinetics and safer therapeutic potential. 

## 5. Materials and Methods

### 5.1. Chemistry

#### 5.1.1. General Methods

Analytical silica gel plates (Merck F254, Sigma-Aldrich, Milan, Italy), preparative silica gel plates (Merck F254, 2 mm), and silica gel 60 (Merck, 70-230 mesh) were used for analytical and preparative TLC, and for column chromatography, respectively. All melting points were determined on a Gallenkamp (U.K) melting point apparatus and are uncorrected. Elemental analyses were performed with a Flash E1112 Thermo Finnigan elemental analyzer for C, H, abd N (Thermo Fisher Scientific, Milan, Italy), and the results were within ±0.4% of the theoretical values. All final compounds revealed purity not less than 95%. The IR spectra were recorded with a Perkin-Elmer Spectrum RX I spectrometer (Perkin-Elmer, Milan, Italy) in Nujol mulls and are expressed in cm^-1^. NMR spectra were recorded on a Bruker Avance 400 spectrometer (Bruker, Milan, Italy) (400 MHz for ^1^H NMR and 100 MHz for ^13^C NMR). The chemical shifts are reported in δ (ppm) and are relative to the central peak of the residual non-deuterated solvent, which was DMSO-d_6_. The following abbreviations are used: s = singlet, d = doublet, t = triplet, q = quartet, m = multiplet, br = broad, Ar = aromatic protons. Compounds **28**–**32** were synthesized as reported in reference [12]. When available, melting point and/or ^1^H NMR values were in accordance to literature data.

#### 5.1.2. Synthesis of the target compounds **1**–**4**, **6**–**13**, and **20**

Sodium hydrogen carbonate (2 mmol) and the suitable halomethyl-derivative (1 mmol) were added to a solution of the suitable mercapto-compound (**28-40** [12,14,18,20,21,22,23], 1 mmol) in anhydrous DMF (1 mL). The reaction mixture was stirred at room temperature (rt) until disappearance of the starting material (TLC monitoring). Then, water was added (25 mL) to precipitate a solid that was collected by filtration and washed with water. The crude product was triturated with Et_2_O (5 mL), collected by filtration and purified.

2-Amino-4-[4-(cyclobutylmethoxy)phenyl]-6-[(1H-imidazol-2-ylmethyl)sulfanyl]pyridine-3,5-dicarbonitrile (**1**)

The crude product was purified by preparative TLC using EtOAc/cyclohexane/MeOH 6:4:1 as eluting system. Yield 20%; mp 214-216 °C. ^1^H NMR (DMSO-d_6_) 11.87 (s, 1H, NH); 8.10 (s, 2H, NH_2_); 7.46 (d, 2H, Ar, J = 7.8 Hz); 7.09 (d, 2H, Ar, J = 7.8 Hz); 7.08 (s, 1H, Ar); 6.96 (s, 1H, Ar); 4.49 (s, 2H, CH_2_); 4.03 (d, 2H, OCH_2_, J = 6.0 Hz); 2.70-2.60 (m, 1H, CH); 2.08-2.07 (m, 2H, CH_2_); 1.91-1.88 (m, 4H, 2CH_2_). IR 3385; 3320; 3217; 2210. Anal. Calc. for C_22_H_20_N_6_OS

2-Amino-6-[(1H-imidazol-2-ylmethyl)sulfanyl]-4-[4-(2-methylpropoxy)phenyl]pyridine-3,5-dicarbonitrile (**2**)

The crude product was purified by preparative TLC using EtOAc/cyclohexane /MeOH 5:5:1 as eluting system. Yield 31%; mp 230-232 °C. ^1^H NMR (DMSO-d_6_) 11.84 (s, 1H, NH); 8.14 (s, 2H, NH_2_), 7.47 (d, 2H Ar, J = 8.7 Hz); 7.10 (d, 2H Ar, J = 8.6 Hz); 7.08 (s, 1H, Ar); 6.85 (s, 1H, Ar); 4.50 (s, 2H, CH_2_); 3.84 (d, 2H, OCH_2_, J = 6.3 Hz); 2.09-2.02 (m, 1H, CH); 1.00 (d, 6H, 2 CH_3_, J = 6.7 Hz); IR 3387; 3315; 3215; 2208. Anal. Calc. for C_21_H_20_N_6_OS

2-Amino-6-[(1H-imidazol-2-ylmethyl)sulfanyl]-4-[4-(prop-2-en-1-yloxy)phenyl]pyridine-3,5-dicarbonitrile (**3**)

The crude product was purified by preparative TLC using DCM/MeOH 9:1 as eluting system. Yield 21%; mp 216-218 °C. ^1^H NMR (DMSO-d_6_) 11.86 (s, 1H, NH); 8.09 (s, 2H, NH_2_); 7.48 (d, 2H Ar, J = 8.5 Hz); 7.12 (d, 2H, Ar, J = 8.6 Hz); 7.07 (s, 1H, Ar); 6.85 (s, 1H, Ar); 6.11-6.04 (m, 1H, CH); 5.40 (d, 1H, CH); 5.30 (d, 1H, CH); 4.62 (d, 2H, OCH_2_, J = 5.2 Hz); 4.48 (s, 2H, CH_2_). Anal. Calc. for C_20_H_16_N_6_OS

2-Amino-6-[(1H-imidazol-2-ylmethyl)sulfanyl]-4-{4-[(2-methylprop-2-en-1-yl)oxy]phenyl} pyridine-3,5-dicarbonitrile (**4**)

The crude product was purified by column chromatography using EtOAc/cyclohexane /MeOH 6:3:1 as eluting system. Yield 36%; mp 224-226 °C. ^1^H NMR (DMSO-d_6_) 11.85 (s, 1H, NH); 8.08 (s, 2H, NH_2_); 7.48 (d, 2H, Ar, J = 8.4 Hz); 7.12 (d, 2H Ar, J = 8.4 Hz); 7.07 (s, 1H, Ar); 6.85 (s, 1H, Ar); 5.09 (s, 1H, CH); 4.99 (d, 1H, CH); 4.56 (s, 2H, CH_2_); 4.49 (s, 2H, OCH_2_); 1.79 (s, 3H, CH_3_); ^13^C NMR (DMSO-d_6_) 167.19; 161.03; 160.95; 159.11; 143.71; 141.67; 131.75; 131.28; 126.93; 116.58; 115.93; 115.80; 113.79; 94.22; 87.00; 72.14; 27.75; 20.32; IR 3404; 3377; 3336; 2210. Anal. Calc. for C_21_H_18_N_6_OS

N-(4-{2-Amino-3,5-dicyano-6-[(1H-imidazol-2-ylmethyl)sulfanyl]pyridin-4-yl}phenyl) acetamide (**6**)

The crude product was purified by column chromatography using EtOAc/cyclohexane /MeOH 8.2:1.5:0.9 as eluting system. Yield 61%; mp 275-278 °C. ^1^H NMR (DMSO-d_6_) 11.84 (s, 1H, NH); 10.22 (s, 1H, NH); 8.11 (s, 2H, NH_2_); 7.73 (d, 2H, Ar, J = 8.3 Hz); 7.46 (d, 2H, ar, J = 8.4 Hz); 7.05 (s, 1H, Ar); 6.86 (s, 1H, Ar); 4.49 (s, 2H, CH_2_); 2.09 (s, 3H, CH_3_); IR 3387; 3211; 2214; 1683. Anal. Calc. for C_19_H_15_N_7_OS

2-Amino-4-(4-chlorophenyl)-6-[(1H-imidazol-2-ylmethyl)sulfanyl]pyridine-3,5-dicarbonitrile (**7**)

Yield 42%; mp 229-231 °C dec (EtOAc). ^1^H NMR (DMSO-d_6_) 11.87 (br s, 1H, NH); 8.2 (br s, 2H, NH_2_); 7.65 (d, 2H, Ar, J = 8.0 Hz); 7.59 (d, 2H, Ar, J = 8.0 Hz); 7.08 (s, 1H, Ar); 6.85 (s, 1H, Ar); 4.50 (s, 2H, CH_2_); IR 3336; 3244; 2220; 1683. Anal. Calc. for C_17_H_11_ClN_6_S

2-Amino-4-(4-fluorophenyl)-6-[(1H-imidazol-2-ylmethyl)sulfanyl]pyridine-3,5-dicarbonitrile (**8**)

Yield 80%; mp 223-225 °C dec (EtOAc). ^1^H NMR (DMSO-d_6_) 11.9 (br s, 1H, NH); 8.2 (br s, 2H, NH_2_); 7.60 (m, 2H, Ar); 7.45 (t, 2H, Ar, J = 8.8 Hz); 7.0 (br s, 2H, Ar); 4.50 (s, 2H, CH_2_); IR 3314; 3145; 2229. Anal. Calc. for C_17_H_11_FN_6_S

2-Amino-6-[(1H-imidazol-2-ylmethyl)sulfanyl]-4-[4-(trifluoromethyl)phenyl]pyridine-3,5-dicarbonitrile (**9**)

Yield 80%; mp 233-235 °C dec (EtOH). ^1^H NMR (DMSO-d_6_) 11.85 (br s, 1H, NH); 8.22 (br s, 2H, NH_2_); 7.97 (d, 2H, Ar, J = 7.8 Hz); 7.79 (d, 2H, Ar, J = 7.8 Hz); 7.08 (s, 1H, Ar); 6.86 (s, 1H, Ar);4.55 (s, 2H, CH_2_); IR 3527; 3185; 2437. Anal. Calc. for C_18_H_11_F_3_N_6_S

2-[(1H-Imidazol-2-il)metiltio]-6-ammino-4(4-metilfenil)piridina-3,5-dicarbonitrile (**10**)

Yield 67%; mp 259-261 °C dec (EtOH). ^1^H NMR (DMSO-d_6_) 11.85 (br s, 1H, NH); 8.14 (br s, 2H, NH_2_); 7.40 (d, 2H, Ar, J = 8.0 Hz); 7.38 (d, 2H, Ar, J = 8.0 Hz); 7.07 (br s, 2H, Ar); 4.50 (s, 2H, CH_2_); 2.39 (s, 3H, CH_3_). IR 3518; 3129; 2213. Anal. Calc. for C_18_H_14_N_6_S

2-Amino-6-[(1H-imidazol-2-ylmethyl)sulfanyl]-4-[4-(methylsulfanyl)phenyl]pyridine-3,5-dicarbonitrile (**11**)

Yield 95%; mp 257-259 °C dec (MeOH). ^1^H NMR (DMSO-d_6_) 11.87 (br s, 1H, NH); 8.12 (br s, 2H, NH_2_); 7.47 (d, 2H, Ar, J = 8.0 Hz); 7.42 (d, 2H, Ar, J = 8.0 Hz); 7.08 (br s, 1H, Ar); 6.85 (br s, 1H, Ar); 4.50 (s, 2H, CH_2_); 2.51 (s, 3H, CH_3_). ^13^C NMR (DMSO-d_6_) 166.65; 160.26; 158.33; 143.00; 142.31; 130.19; 129.43; 125.69; 115.81; 93.54; 86.35; 27.23; 14.47. IR 3420; 3390; 2223. Anal. Calc. for C_18_H_14_N_6_S_2_

2-Amino-4-benzyl-6-[(1H-imidazol-2-ylmethyl)sulfanyl]pyridine-3,5-dicarbonitrile (**12**)

Yield 70%; mp 246-248 °C dec (EtOH). ^1^H NMR (DMSO-d_6_) 11.82 (br s, 1H, NH); 8.14 (br s, 2H, NH_2_); 7.34 (d, 2H, Ar, J = 8.0 Hz); 7.24 (m, 3H, Ar); 6.95 (br s, 2H, Ar); 4.47 (s, 2H, CH_2_); 4.08 (s, 2H, CH_2_). ^13^C NMR (DMSO-d_6_) 166.73; 160.33; 159.10; 142.89; 136.61; 129.38; 128.78; 127.64; 115.61; 93.99; 86.95; 38.84; 27.15; 14.47. IR 3431; 3357; 2377. Anal. Calc. for C_18_H_14_N_6_S

2-Amino-6-[(1H-imidazol-2-ylmethyl)sulfanyl]-4-methylpyridine-3,5-dicarbonitrile (**13**)

Yield 66%; mp 267-269 °C dec (EtOH). ^1^H NMR (DMSO-d_6_) 11.87 (br s, 1H, NH); 8.12 (br s, 2H, NH_2_); 7.02 (br s, 2H, Ar); 4.51 (s, 2H, CH_2_), 2.43 (s, 3H, CH_3_). IR 3455; 3363; 2473. Anal. Calc. for C_12_H_10_N_6_S

Methyl{[6-amino-3,5-dicyano-4-(4-methoxyphenyl)pyridin-2-yl]sulfanyl}acetate (**20**)

Yield 80%; mp 218-220 °C (EtOH). ^1^H NMR (DMSO-d_6_) 7.96 (br s, 2H, NH_2_); 7.51 (d, 2H, Ar, J = 8.8 Hz); 7.12 (d, 2H, Ar; J = 8.8 Hz)); 4.20 (s, 2H, CH_2_), 3.84 (s, 3H, CH_3_), 3.70 (s, 3H, CH_3_). IR 3426; 3397; 3325; 3224; 2209. Anal. Calc. for C_17_H_14_N_4_O_3_S

#### 5.1.3. Synthesis of the Target Compounds **14**–**17**

The suitable commercially available amino-derivative (1-(1H-imidazol-2-yl)methanamine, for compound **14**; ethanolamine, for **15**; 2-(4-methoxyphenyl)ethanamine, for **16**; or imidazole (for compound **17** (1.18 mmol), and triethylamine (1.18 mmol) were sequentially added to a solution of the 2-chloro-derivative **42** [27] (0.59 mmol) in a mixture THF/EtOH 2:1 (7.5 mL). The reaction mixture was heated at reflux until the disappearance of the starting material (TLC monitoring) and then cooled at rt. After dilution with water (20 mL), a solid precipitated was collected and washed with water and Et_2_O.

2-Amino-6-[(1H-imidazol-2-ylmethyl)amino]-4-phenylpyridine-3,5-dicarbonitrile (**14**)

Yield 57%; mp 208-210 °C (EtOH). ^1^H NMR (DMSO-d_6_) 11.64 (br s, 1H, NH); 8.39 (br s, 2H, NH_2_); 7.88 (t, 1H, NH, J = 5.5 Hz); 7.61-7.45 (m, 5H, Ar); 7.04 (br s, 1H, Ar); 6.83 (br s, 1H, Ar);4.56 (d, 2H, CH_2_, J = 5.6 Hz). IR 3360; 3322; 3186; 2207. Anal. Calc. for C_17_H_13_N_7_

2-Amino-6-[(2-hydroxyethyl)amino]-4-phenylpyridine-3,5-dicarbonitrile (**15**) 

Yield 64%; mp 230-231 °C (EtOH). ^1^H NMR (DMSO-d_6_) 7.55-7.53 (m, 3H, Ar); 7.47-7.45 (m, 2H, ar); 7.37 (br s, 2H, NH_2_); 7.20 (t, 1H, NH, J = 5.1 Hz); 4.74 (t, 1H, OH, J = 5.2 Hz); 3.54 (m, 2H, CH_2_); 3.47 (m, 2H, CH_2_). IR 3472; 3345; 3223; 2204. Anal. Calc. for C_15_H_13_N_5_O

2-Amino-6-{[2-(4-methoxyphenyl)ethyl]amino}-4-phenylpyridine-3,5-dicarbonitrile (**16**)

Yield 80%; mp ˃ 300 °C (EtOH/DMF). ^1^H NMR (DMSO-d_6_) 7.54-7.53 (m, 3H, Ar); 7.51-7.45 (m, 3H, 2 Ar + NH); 7.39 (br s, 2H, NH_2_); 7.20 (d, 2H, Ar, J = 8.4 Hz); 6.87 (d, 2H, Ar, J = 8.4 Hz); 3.73 (s, 3H, CH_3_); 3.57-3.51 (m, 2H, CH_2_); 2.80 (t, 2H, CH_2_, J = 8.0 Hz). IR 3451; 3342; 3244; 2201. Anal. Calc. for C_22_H_19_N_5_O

2-Amino-6-(1H-imidazol-1-yl)-4-phenylpyridine-3,5-dicarbonitrile (**17**)

Yield 77%; mp 253-255 °C (EtOH). ^1^H NMR (DMSO-d_6_) 8.40 (br s, 2H, NH_2_), 8.36 (s, 1H, Ar), 7.81 (s, 1H, Ar), 7.63 (m, 5H, Ar), 7.18 (s, 1H, Ar). IR 3399; 2220; 1681;1466. Anal. Calc. for C_16_H_10_N_6_

#### 5.1.4. Synthesis of 2-amino-4-phenylpyridine-3,5-dicarbonitrile (**18**)

Ten per cent Pd/C (0.1 g) was added to a warm solution of the 2-chloro-derivative **42** [27] (0.98 mmol, 0.25 g) in absolute EtOH (50 mL). The mixture was hydrogenated in a Parr apparatus, at 30 Psi for 6 h. The catalyst was filtered off and the solvent was distilled to a small volume under reduced pressure to yield a yellow solid, which was collected and washed with Et_2_O. Yield 33%; mp 244-246 °C (2- Propanol). ^1^H NMR (DMSO-d_6_) 8.66 (s, 1H, ar), 8.10 (br s, 2H, NH_2_), 7.52-7.60 (m, 5H, ar). IR 3328; 3140; 2224; 1660. Anal. Calc. for C_13_H_8_N_4_

##### 5.1.5. 2-Amino-6-[(2-hydroxyethyl)amino]-4-(4-methoxyphenyl)pyridine-3,5-dicarbonitrile (**19**)

A suspension of the 6-phenylsulfanyl derivative **27** [24] (1.81 mmol) and an excess of ethanolamine (7.25 mmol) in anhydrous DMF (7.8 mL) was heated at 100 °C for 5h. After cooling at rt, water (30 mL) was added. A solid precipitated, which was collected by filtration and washed with water and Et_2_O. Yield 90%; mp 248-250 °C (EtOH). ^1^H NMR (DMSO-d_6_) 7.43 (d, 2H, Ar, J = 8.8 Hz); 7.32 (br s, 2H, NH_2_); 7.14 (t, 1H, NH), 7.09 (d, 2H, Ar, J = 8.8 Hz)); 4.74 (t, 1H, OH), 3.84 (s, 3H, CH_3_), 3.56-3.53 (m, 2H, CH_2_), 3.50-3.47 (m, 2H, CH_2_). IR 3331; 3226; 2204. Anal. Calc. for C_16_H_15_N_5_O_2_

#### 5.1.6. Synthesis of 4-substituted-2-amino-6-(phenylsulfanyl)pyridine-3,5-dicarbonitriles **21**–**27** [18,24–26]

A suspension of the suitable aldehyde (8.9 mmol), malononitrile (20.8 mmol), thiophenol (10.4 mmol), and basic alumina (0.019 mol) in water (20 mL) was stirred at 100 °C until the disappearance of the starting material (TLC monitoring). After cooling at rt, the aqueous layer was separated by decantation from the yellow sticky mass. The residue was treated with a mixture EtOH/Et_2_O 1:2 (3 mL) yielding a solid which was collected by filtration and extracted with boiling EtOAc (100 mLx2). After distillation of the combined and dried (Na_2_SO_4_) organic layers, the crude product was triturated with a little of Et_2_O/EtOH 1:1 and collected by filtration. The compounds were used without further purification unless otherwise stated.

2-Amino-4-(4-chlorophenyl)-6-(phenylsulfanyl)pyridine-3,5-dicarbonitrile (**21**) [24]

Yield 75%; mp 236-237 °C (lit mp 236-237 °C)

2-Amino-4-(4-fluorophenyl)-6-(phenylsulfanyl)pyridine-3,5-dicarbonitrile (**22**) [25]

Yield 78%; mp 220-222 °C (lit mp 221-223 °C)

2-Amino-6-(phenylsulfanyl)-4-[4-(trifluoromethyl)phenyl]pyridine-3,5-dicarbonitrile (**23**) [18]

Yield 64%; mp 199-201 °C (EtOH). ^1^HNMR (DMSO-d_6_) 7.98 (d, 2H, J = 7.8 Hz); 7.92 (br s, 2H, NH_2_); 7.82 (d, 2H, Ar, J = 7.8 Hz); 7.63-7.61 (m, 2H, Ar); 7.52-7.51 (m, 3H, Ar). IR 3320; 3211; 2243. Anal. Calc. for C_20_H_11_F_3_N_4_S

2-Amino-4-(4-methylphenyl)-6-(phenylsulfanyl)pyridine-3,5-dicarbonitrile (**24**) [24]

Yield 74%; mp 220-221 °C (lit mp 215-216 °C).

2-Amino-4-[4-(methylsulfanyl)phenyl]-6-(phenylsulfanyl)pyridine-3,5-dicarbonitrile (**25**) [26]

Yield 75%; mp 244-246 °C (lit mp 254-256 °C).

2-Amino-4-benzyl-6-(phenylsulfanyl)pyridine-3,5-dicarbonitrile (**26**)

Yield 70%; mp 247-249 °C (MeOH). ^1^HNMR (DMSO-d_6_) 7.81 (br s, 2H, NH_2_); 7.60-7.57 (m, 2H, Ar); 7.47-7.50 (m, 3H, Ar); 7.39-7.35 (m, 2H, Ar); 7.29-7.25 (m, 3H, Ar); 4.12 (s, 2H, CH_2_). IR 3329; 3225; 2198. Anal. Calc. for C_20_H_14_N_4_S

2-Amino-4-(4-methoxyphenyl)-6-(phenylsulfanyl)pyridine-3,5-dicarbonitrile (**27**) [24]

Yield 66%; mp 252-254 °C (lit mp 254-255 °C). 

#### 5.1.7. Synthesis of 2-amino-4-substituted-6-sulfanylpyridine-3,5-dicarbonitriles **33**–**40** [14,18,20,21] 

An excess of anhydrous sodium sulfide (33 mmol) was added to a stirred solution of the suitable sulfanyl derivative **21-27** [18,24,25,26] (10 mmol) in anhydrous DMF (1 mL), maintained at rt and under nitrogen atmosphere. The reaction mixture was heated at 80 °C until the disappearance of the starting material (TLC monitoring). Then, 6N HCl (25 mL) was drop by drop added to obtain a precipitate, which was collected by filtration and washed with water (20 mL) and Et_2_O (5 mL). Compounds were used without further purification unless otherwise stated.

2-Amino-4-(4-chlorophenyl)-6-sulfanylpyridine-3,5-dicarbonitrile (**33**) [20,21]

Yield 80%; mp 202-204 °C (lit mp 110°C). ^1^HNMR (DMSO-d_6_) 13.12 (br s, 1H, SH); 8.01 (sl, 2H, NH_2_); 7.65 (d, 2H, Ar, J = 8.0 Hz); 7.58 (d, 2H, Ar, J = 8.0 Hz). IR 3320; 3192; 2220. Anal. Calc. for C_13_H_7_ClN_4_S

2-Amino-4-(4-fluorophenyl)-6-sulfanylpyridine-3,5-dicarbonitrile (**34**) [18]

Yield 86%; mp 234-236 °C. ^1^HNMR (DMSO-d_6_) 13.03 (br s, 1H, SH); 8.01(br s, 2H, NH_2_); 7.58-7.52 (m, 2H, Ar); 7.38 (m, 2H, Ar). IR 3319; 3182; 2224.

2-Amino-6-sulfanyl-4-[4-(trifluoromethyl)phenyl]pyridine-3,5-dicarbonitrile (**35**) [18] 

Yield 90%; mp 212-214 °C (MeOH). ^1^HNMR (DMSO-d_6_) 13.15 (br s, 1H, SH); 8.07 (d, 2H, Ar, J = 8.0 Hz); 7.8 (d, 2H, Ar, J = 8.0 Hz). IR 3310; 3193; 2225.

2-Amino-4-(4-methylphenyl)-6-sulfanylpyridine-3,5-dicarbonitrile (**36**) [18] 

Yield 83%; mp 228-230 °C. ^1^HNMR (DMSO-d_6_) 13.03 (br s, 1H, SH); 8.0 (br s, 2H, NH_2_); 7.41-7.37 (m, 4H, Ar). IR 3322; 3190; 2215.

2-Amino-4-[4-(methylsulfanyl)phenyl]-6-sulfanylpyridine-3,5-dicarbonitrile (**37**)

Yield 82%; mp 188-190 °C (EtOH). ^1^HNMR (DMSO-d_6_) 13.1 (br s, 1H, SH); 8.03 (br s, 2H, NH_2_), 7.46 (d, 2H, Ar, J = 8.0 Hz); 7.41 (d, 2H, Ar J = 8.0 Hz); 2.51 (s, 3H, CH_3_). IR 3311; 3195; 2222. Anal. Calc. for C_14_H_10_N_4_S_2_

2-Amino-4-benzyl-6-sulfanylpyridine-3,5-dicarbonitrile (**38**)

Yield 80%; mp 231-233 °C (EtOH). ^1^HNMR (DMSO-d_6_) 12.96 (br s, 1H, SH); 7.82 (br s, 2H); 7.37-7.35 (m, 2H, Ar); 7.30-7.28 (m, 3H, Ar); 4.01 (s, 2H, CH_2_). IR 3319; 3280; 2229. Anal. Calc. for C_14_H_10_N_4_S

2-Amino-4-(4-methoxyphenyl)-6-sulfanylpyridine-3,5-dicarbonitrile (**40**) [14]

Yield 80%; mp 261-262 °C (EtOH). ^1^H NMR (DMSO-d_6_) 7.96 (br s, 2H, NH_2_); 7.52 (d, 2H, Ar, J = 8.8 Hz); 7.13 (d, 2H, Ar; J = 8.8 Hz)); 3.85 (s, 3H, CH_3_). IR 3289; 3182; 2213.

#### 5.1.8. Synthesis of 2-amino-4-methyl-6-sulfanylpyridine-3,5-dicarbonitrile (**39**) [22,23]

Compound **41** [22] (23 mmol) and 2-cyanothioacetamide (22.3 mmol) were consequentially added to an ethanolic solution of EtONa (570 mg of Na in 50 mL absolute EtOH). The reaction mixture was heated at reflux for 4 h. After evaporation of the solvent at small volume, a yellow solid precipitated which was collected under suction, washed with water and recrystallized. Yield 90%; mp 226-228 °C (EtOH) (lit mp 225 °C [23]; lit mp 120-123 °C [22]). ^1^H NMR (DMSO-d_6_) 12.94 (br s, 1H, SH); 7.1 (br s, 2H, NH_2_); 2.25 (s, 3H, CH_3_). IR 3338; 3253; 2248.

#### 5.1.9. Synthesis of 2-amino-6-chloro-4-phenylpyridine-3,5-dicarbonitrile (**42**) [27] 

Malononitrile (15.2 mmol) was portion-wise added to a solution of triethyl orthobenzoate (8mmol) and pyridine (0.6 mL) at 100 °C. Heating was continued for 6 h, and after cooling at 0 °C, 12 M HCl (2.7 mL) was drop by drop added to the mixture. The suspension was heated at 100 °C for 2 h, and the resulting precipitate was then collected by filtration and suspended in NaHCO_3_ saturated solution (5 mL). After stirring at room temperature for 30 min, the solid was filtered by suction and washed with water. Yield 61%; mp ˃ 300 °C (MeOH) (lit mp ˃ 250 °C). ^1^H NMR (DMSO-d_6_) 8.41 (br s, 2H, NH_2_), 7.61-7.57 (m, 5H, Ar); IR 3356; 3179; 2225;1660;1564.

### 5.2. Pharmacological Assays

#### 5.2.1. Cell Culture and Membrane Preparation.

CHO cells transfected with hA_1_, hA_2A_, hA_2B_, and hA_3_ ARs were grown adherently and maintained in Dulbecco’s modified Eagle’s medium (Sigma-Aldrich, Milan, Italy) with nutrient mixture F12, containing 10% fetal calf serum, penicillin (100 U/mL), streptomycin (100 μg/mL), l-glutamine (2 mM) and geneticin (G418; 0.2 mg/mL) at 37°C in 5% CO_2_/95% air until the use in cAMP assays [42]. For membrane preparation, the culture medium was removed, and the cells were washed with phosphate-buffered saline and scraped off T75 flasks in ice-cold hypotonic buffer (5 mM Tris HCl, 1 mM EDTA, pH 7.4). The cell suspension was homogenized with a Polytron, centrifuged for 30 min at 40000 *g* at 4°C, and the resulting membrane pellet was used for competition binding experiments [42].

#### 5.2.2. Competition Binding Experiments

All synthesized compounds have been tested for their affinity to hA_1_, hA_2A_, and hA_3_ ARs. Competition experiments to hA_1_ ARs were carried out incubating 1 nM [^3^H]-8-cyclopentyl-1,3-dipropylxanthine ([^3^H]-DPCPX) with membrane suspension (50 μg of protein/100 μL) and different concentrations of the examined compounds at 25°C for 90 min in 50 mM Tris HCl, pH 7.4. Non-specific binding was defined as binding in the presence of 1 μM DPCPX and was always < 10% of the total binding [42]. Inhibition experiments to hA_2A_ ARs were performed incubating the radioligand [^3^H]-ZM241385 (4-(2-[7-Amino-2-(2-furyl)[1,2,4]triazolo[2,3-a][1,3,5]triazin-5-ylamino]ethyl)phenol) (1 nM) with the membrane suspension (50 μg of protein/100 μL) and different concentrations of the examined compounds for 60 min at 4°C in 50 mM Tris HCl (pH 7.4), 10 mM MgCl_2_. Non-specific binding was determined in the presence of ZM241385 (1 μM) and was about 20% of the total binding [43]. Competition binding experiments to A_3_ ARs were carried out incubating the membrane suspension (50 μg of protein/100 μL) with 0.5 nM [^125^I]-*N*^6^-(4-aminobenzyl)-*N*-methylcarboxamidoadenosine ([^125^I]-ABMECA) in the presence of different concentration of the examined compounds for an incubation time of 120 min at 4°C in 50 mM Tris HCl (pH 7.4), 10 mM MgCl_2_, and 1 mM EDTA. Non-specific binding was defined as binding in the presence of 1μM ABMECA and was always < 10% of the total binding [44]. Bound and free radioactivity were separated by filtering the assay mixture through Whatman GF/B glass fiber filters using a Brandel cell harvester (Brandel Instruments, Unterföhring, Germany). The filter bound radioactivity was counted by Packard Tri Carb 2810 TR scintillation counter (Perkin Elmer, Boston, MA, USA).

#### 5.2.3. Cyclic AMP Assays.

CHO cells transfected with hAR subtypes were washed with phosphate-buffered saline, detached with trypsin and centrifuged for 10 min at 200 g. Cells were seeded in 96-well white half-area microplate (Perkin Elmer, Boston, USA) in a stimulation buffer composed of Hank Balanced Salt Solution (Sigma-Aldrich, Milan, Italy), 5 mM HEPES, 0.5 mM Ro 20-1724, and 0.1% BSA. cAMP levels were then quantified by using the AlphaScreen cAMP Detection Kit (Perkin Elmer) following the manufacturer’s instructions [45]. At the end of the experiments, plates were read with the Perkin Elmer EnSight Multimode Plate Reader.

#### 5.2.4. Data Analysis

The protein concentration was determined according to a Bio-Rad method with bovine albumin as a standard reference. Inhibitory binding constant (*K*i) values were calculated from those of IC_50_ according to Cheng & Prusoff equation *K*i = IC_50_/(1+[C*]/*K*_D_*), where [C*] is the concentration of the radioligand and *K*_D_* its dissociation constant [44]. *K*_i_ and IC_50_ values were calculated by non-linear regression analysis using the equation for a sigmoid concentration-response curve (Graph-PAD Prism, San Diego, CA, USA).

### 5.3. Molecular Modelling

Homology modelling and energy minimization studies were carried out using MOE (Molecular Operating Environment, C.C.G., Inc., 1255 University St., Suite 1600, Montreal, Quebec, Canada, H3B 3X3, version 2019.0101) suite [30]. A homology model of the hA_2B_ AR was built using the X-ray structure of the hA_2A_ AR in complex with Ado as template (pdb code: 2YDO; 3.0-Å resolution [29]). A multiple alignment of the hAR primary sequences was built within MOE as preliminary step. The boundaries identified from the used X-ray crystal structure of hA_2A_ AR were then applied for the corresponding sequences of the TM helices of the hA_2B_ AR. The missing loop domains were built by the loop search method implemented in MOE. Once the heavy atoms were modelled, all hydrogen atoms were added, and the protein coordinates were then minimized with MOE using the AMBER10 force field until the Root Mean Square (RMS) gradient of the potential energy was less than 0.05 kJ mol^−1^ Å^−1^. Reliability and quality of the model were checked using the Protein Geometry Monitor application within MOE, which provides a variety of stereochemical measurements for inspection of the structural quality in a given protein, like backbone bond lengths, angles and dihedrals, Ramachandran φ-ψ dihedral plots, and quality of side chain rotamer and non-bonded contact. The cryo-EM structure of the agonist-bound hA_1_ AR (pdb code: 6D9H; 3.6-Å resolution [28]) was added of hydrogen atoms and energetically minimized following the same protocol above described; the coordinates of the heavy atoms were kept fixed in this task.

All compound structures were docked into the binding site of the hA_2B_ and hA_1_ ARs structures using as docking tools the Induced Fit docking protocol of MOE and the genetic algorithm docking tool of CCDC Gold [31]. The Induced Fit docking protocol of MOE is divided into the following several stages:

- *Conformational Analysis of ligands*. The algorithm generated conformations from a single 3D conformation by conducting a systematic search. In this way, all combinations of angles were created for each ligand.
- *Placement*. A collection of poses was generated from the pool of ligand conformations using Alpha Triangle placement method. Poses were generated by superposition of ligand atom triplets and triplet points in the receptor binding site. The receptor site points are alpha sphere centres which represent locations of tight packing. At each iteration, a random conformation was selected, a random triplet of ligand atoms and a random triplet of alpha sphere centres were used to determine the pose.
- *Scoring*. Poses generated by the placement methodology were scored using the Alpha HB scoring function, which combines a term measuring the geometric fit of the ligand to the binding site and a term measuring hydrogen bonding effects.
- *Induced Fit*. The generated docking conformations were subjected to energy minimization within the binding site and the protein sidechains are included in the refinement stage. In detail, the protein backbone is set as rigid while the side chains are not set to “free to move” but are set to “tethered”, where an atom tether is a distance restraint that restrains the distance not between two atoms but between an atom and a fixed point in space.
- *Rescoring*. Complexes generated by the Induced Fit methodology stage were scored using the Alpha HB scoring function. Gold tool was used with default efficiency settings through MOE interface, by selecting GoldScore as scoring function [31].

## Figures and Tables

**Table 1:**
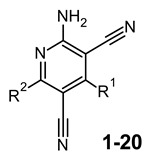
Binding affinities (*K_i_*) at hA_1_, hA_2A_, and hA_3_ ARs and potencies (EC_50_) at hA_2B_ ARs.

			cAMP Assays	Binding Experiments ^a^		
			EC_50_ (nM) ^b^ Efficacy, ^c^ %	*K*_i_ (nM) or I%		
			hA_2B_	hA_1_^d^	hA_2A_^e^	hA_3_^f^
	R^1^	R^2^				
**1**	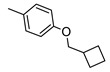	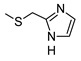	˃1000 (12%)	25%	4%	574 ± 46
**2**	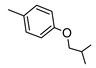	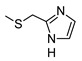	227 ± 21 (36%)	26%	1%	1%
**3**	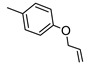	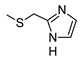	27 ± 3 (56%)	16 ± 2	17%	8%
**4**	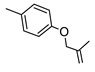	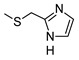	˃1000 (14%)	77 ± 6	1%	8%
**5^g^**	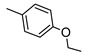	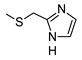	11.7 ± 1.2 (62%)	8.21 ± 0.73	221 ± 19	85 ± 6
**6**	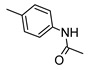	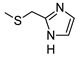	12 ± 1 (100%)	83 ± 7	38 ± 3	48 ± 4
**7**	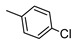	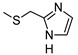	109 ± 12 (32%)	30 ± 4	865 ± 68	850 ± 62
**8**	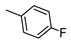	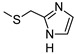	18 ± 2 (76%)	0.78 ± 0.08	139 ± 12	410 ± 38
**9**	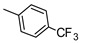	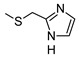	˃1000 (4%)	419 ± 32	32%	21%
**10**	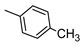	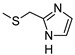	78 ± 6 (78%)	5.32 ± 0.42	47 ± 4	212 ± 19
**11**	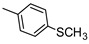	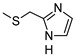	36 ± 3 (30%)	8.32 ± 0.78	227 ± 21	388 ± 29
**12**	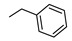	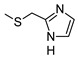	260 ± 24 (44%)	31%	8%	1%
**13**	CH_3_	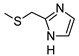	˃1000 (1%)	74 ± 8	331 ± 32	510 ± 53
**14**	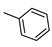	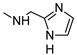	˃1000 (1%)	140 ± 12	115 ± 10	19%
**15**	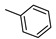	NHCH_2_CH_2_OH	˃1000 (1%)	943 ± 87	1%	1%
**16**	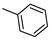	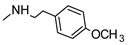	˃1000 (1%)	1%	1%	1%
**17**	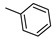	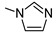	˃1000 (1%)	27%	1%	25%
**18**	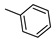	H	˃1000 (1%)	15%	16%	5%
**19**	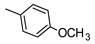	NHCH_2_CH_2_OH	˃1000 (1%)	14%	2%	15%
**20**	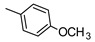	SCH_2_COOCH_3_	˃1000 (1%)	987 ± 81	1%	5%
**P453 ^g^**	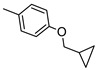	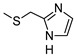	9.52 ± 0.91 (70%)	235 ± 24	764 ± 72	474 ± 45
**LUF5833 ^h^**	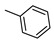	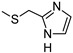	19 ± 7 (81%)	2.4 ± 1	28 ± 1	171 ± 109

^a^*K*_i_ values are means ± SEM of four separate assays each performed in triplicate. Percentage of inhibition (I%) is determined at 1µM concentration of the tested compounds. ^b^ EC_50_ values are means ± SEM of four separate assays each performed in triplicate. ^c^ Efficacy of the tested compound at 1µM concentration, in comparison with NECA (1µM = 100%). ^d^ Displacement of specific [^3^H]DPCPX competition binding to hA_1_CHO cells. ^e^ Displacement of specific [^3^H]ZM241385 competition binding to hA_2A_CHO cells. ^f^ Displacement of specific [^125^I]AB-MECA competition binding to hA_3_CHO cells. ^g^ Reference [12]. ^h^ Reference [14].

**Table 2:** Modulation of Forskolin-stimulated cAMP levels of selected amino-3,5-dicyanopyridine derivatives on cyclic cAMP assay in hA_1_ CHO cells.^a.^

compd	Efficacy, ^b^ %	EC_50_ (nM) ^c^
**3**	39 ± 4	19.6 ± 1.7
**8**	34 ± 3	1.34 ± 0.11
**11**	52 ± 4	8.87 ± 0.64
**DPCPX**	100 ± 9	1.52 ± 0.12

^a^ Potency values (EC_50_) are expressed as means ± SEM of four independent cAMP experiments, each performed in triplicate. The compounds have been tested on Forskolin at 5 µM concentration. ^b^ Efficacy of the novel compounds were normalized by using the efficacy value of the reference compound DPCPX (set at 100%) that showed the higher capability to increase cAMP production. ^c^ Potency of the novel compounds to increase Forskolin-stimulated cAMP levels.

## Supplementary Materials

The following are available online at https://www.mdpi.com/1424-8247/12/4/159/s1: Table S1. Combustion analysis data of the newly synthesized compounds **1**–**4**, **6**–**20**, **26**, **37**, **38**, and derivative **33**. Table S2. Calculated molecular properties and ADMET parameters for compound **3**.

## Abbreviations

ABMECA	*N*^6^-(4-Aminobenzyl)-N-methylcarboxamidoadenosine
AC	Adenylate Cyclase
ADMET	Absorption, Distribution, Metabolism, Excretion, Toxicity
Ado	Adenosine
AR	Adenosine Receptor
BSA	Bovine Serum Albumin
CCPA	2-Chloro-N^6^-cyclopentyladenosine
CHO	Chinese Hamster Ovary
CPA	*N*^6^-Cyclopentyladenosine
DPCPX	8-Cyclopentyl-1,3-dipropylxanthine
EL	Extracellular loop
EtOAc	Ethyl Acetate
DMF	Dimethylformamide
DMSO	Dimethylsulfoxide
EDTA	Ethylendiaminotetracetic Acid
HEPES	4-(2-Hydroxyethyl)-1-piperazine-1-ethane sulfonic Acid
MOE	Molecular operating environment;
NECA	5′-(*N*-Ethylcarboxamido)adenosine
rt	room temperature
SARs	Structure-Activity Relationships
THF	Tetrahydrofuran
TM	Transmembrane

## References

[1] Borea P.A., Gessi S., Merighi S., Varani K. (2016). Adenosine as a multisignalling guardian angel in human diseases: When, where and how does it exert its protective effects?. Trends Pharmacol. Sci..

[2] Borea P.A., Gessi S., Merighi S., Vincenzi F., Varani K. (2018). Pharmacology of adenosine receptors: The state of the art. Physiol. Rev..

[3] Chen J.F., Eltzschig H.K., Fredholm B.B. (2013). Adenosine receptors as drug targets—what are the challenges?. Nature Rev. Drug Discov..

[4] Müller C.E., Jacobson K.A. (2011). Recent developments in adenosine receptor ligands ad their potential as novel drugs. Biochim. Biophys. Acta.

[5] Dhalla A.K., Chisholm J.W., Reaven G.M., Belardinelli L. (2009). A1 adenosine receptor: Role in diabetes and obesity. Handb. Exp. Pharmacol..

[6] Koupenova M., Ravid K. (2013). Adenosine, adenosine receptors and their role in glucose homeostasis and lipid metabolism. J. Cell. Physiol..

[7] Eisenstein A., Patterson S., Ravid K. (2015). The many faces of the A_2B_ adenosine receptor in cardiovascular and metabolic diseases. J. Cell. Physiol..

[8] Wojcik M., Zieleniak A., Wozniak L.A. (2010). New insight into A_1_ adenosine receptors in diabetes treatment. Curr. Pharm. Design.

[9] Gessi S., Merighi S., Fazzi D., Stefanelli A., Varani K., Borea P.A. (2011). Adenosine receptor targeting in health and disease. Expert Opin. Investig. Drug..

[10] Johansson S.M., Lindgren E., Yang J.N., Herling A.W., Fredholm B.B. (2008). Adenosine A_1_ receptors regulate lipolysis and lipogenesis in mouse adipose tissue-interactions with insulin. Eur. J. Pharmacol..

[11] Rosentreter U., Henning R., Bauser M., Krämer T., Vaupel A., Hübsch W., Dembowsky K., Salcher-Schraufstätter O., Stasch J.P., Krahn T. (2001). Substituted 2-Thio-3,5-Dicyano-4-Aryl-6-Aminopyridines and the use Thereof as Adenosine Receptor Ligands. WO/2001/025210.

[12] Betti M., Catarzi D., Varano F., Falsini M., Varani K., Vincenzi F., Dal Ben D., Lambertucci C., Colotta V. (2018). The aminopyridine-3,5-dicarbonitrile core for the design of new non-nucleoside-like agonists of the human adenosine A_2B_ receptor. Eur. J. Med. Chem..

[13] Fusco I., Cherchi F., Catarzi D., Colotta V., Varano F., Pedata F., Pugliese A.M., Coppi E. (2019). Functional characterization of a novel adenosine A_2B_ receptor agonist on short-term plasticity and synaptic inhibition during oxygen and glucose deprivation in the rat CA1 hippocampus. Brain Res. Bull..

[14] Beukers M.W., Chang L.C.W., von Frijtag Drabbe Kunzel J.K., Mulder-Krieger T., Spanjersberg R.F., Brussee J., IJzerman A.P. (2004). New, non-adenosine, high-potency agonists for the human adenosine A_2B_ receptor with an improved selectivity profile compared to the reference agonist N-ethylcarboxamidoadenosine. J. Med. Chem..

[15] Betti M., Catarzi D., Varano F., Falsini M., Varani K., Vincenzi F., Pasquini S., di Cesare Mannelli L., Ghelardini C., Lucarini E. (2019). Modifications on the amino-3,5-dicyanopyridine core to obtain multifaceted adenosine receptor ligands with antineuropathic activity. J. Med. Chem..

[16] Baraldi P.G., Tabrizi M.A., Fruttarolo F., Romagnoli R., Preti D. (2009). Recent improvement in the development of A_2B_ adenosine receptor agonists. Purinergic Signal..

[17] Krahn T., Krämer T., Rosentreter U., Downey J.M., Solenkova N. (2006). Use of substituted 2-thio-3,5-dicyano-4-phenyl-6-aminopyridines for the treatment of reperfusion injury and reperfusion damage. WO 2006099958 A1.

[18] Chang L.C.W., von Frijtag Drabbe Künzel J.K., Mulder-Krieger T., Spanjersberg R.F., Roerink S.F., van den Hout G., Beukers M.W., Brussee J., IJzerman A.P. (2005). A series of ligand displaying a remarkable agonistic-antagonistic profile at the adenosine A_1_ receptor. J. Med. Chem..

[19] Meibom D., Albrecht-Küpper B., Diedrichs N., Hübsch W., Kast R., Krämer T., Krenz U., Lerchen H.-J., Mittendorf J., Nell P.G. (2017). Neladenoson bialanate hydrochloride: A prodrug of a partial adenosine A_1_ receptor agonist for the chronic treatment of heart disease. ChemMedChem..

[20] El-Torgoman A.M., El-Kousy S.M., El-Shahat K.Z. (1987). Nitriles in heterocyclic synthesis: The reaction of 2-(thiocarbamoyl)cinnamonitriles with active methylene reagents. Z. Naturforsch. B Chem. Sci..

[21] Alinaghizadeh F., Zahedifar M., Seifi M., Sheibani H. (2016). Cascade synthesis of thieno[2,3-b]pyridines by using intramolecular cyclization reactions of 3-cyano-2-(organylmethylthio)pyridines. J. Braz. Chem. Soc..

[22] Ibrahim D.A., Ismail N.S.M. (2011). Design, synthesis and biological study of novel pyrido[2,3-d]pyrimidine as anti-proliferative CDK2 inhibitors. Eur. J. Med. Chem..

[23] Geies A.A., Kamal El-Dean A.M., Abdel Monem M.I. (1992). Reinvestigation of the reaction of arylidenemalonitriles with cyanothioacetamide: New approach for the synthesis of pyridine derivatives. Z. Naturforsch. B..

[24] Kambe S., Saito K., Sakurai A., Midorikawa H. (1981). Synthetic studies using α,β-unsaturated nitriles: Facile synthesis of pyridine derivatives. Synthesis.

[25] Guo K., Thompson M.J., Reddy T.R.K., Mutter R., Chen B. (2007). Mechanistic studies leading to a new procedure for rapid, microwave assisted generation of pyridine-3,5-dicarbonitrile libraries. Tetrahedron.

[26] Ranu B.C., Jana R., Sowmiah S. (2007). An improved procedure for the three-component synthesis of highly substituted pyridines using ionic liquid. J. Org.Chem..

[27] Murray T.J., Zimmerman S.C., Kolotuchin S.V. (1995). Synthesis of heterocyclic compounds containing three contiguous hydrogen bonding sites in all possible arrangements. Tetrahedron.

[28] Draper-Joyce C.J., Khoshouei M., Thal D.M., Liang Y.L., Nguyen A.T.N., Furness S.G.B., Venugopal H., Baltos J.A., Plitzko J.M., Danev R. (2018). Structure of the adenosine-bound human adenosine A_1_ receptor-Gi complex. Nature.

[29] Lebon G., Warne T., Edwards P.C., Bennett K., Langmead C.J., Leslie A.G., Tate C.G. (2011). Agonist-bound adenosine A_2A_ receptor structures reveal common features of GPCR activation. Nature.

[30] Molecular Operating Environment.

[31] Jones G., Willett P., Glen R.C., Leach A.R., Taylor R. (1997). Development and validation of a genetic algorithm for flexible docking. J. Mol. Biol..

[32] Rosentreter U., Krämer T., Shimada M., Hübsch W., Diedrichs N., Krahn T., Henninger K., Stasch J.P. (2003). Substituted 2-thio-3,5-dicyano-4-phenyl-6-aminopyridines and their use as adenosine receptor-selective ligands. WO/2003/008384.

[33] Thimm D., Schiedel A.C., Sherbiny F.F., Hinz S., Hochheiser K., Bertarelli D.C.G., Maass A., Müller C.E. (2013). Ligand-Specific Binding and Activation of the Human Adenosine A2B Receptor. Biochemistry.

[34] Dal Ben D., Buccioni M., Lambertucci C., Thomas A., Volpini R. (2013). Simulation and comparative analysis of binding modes of nucleoside and non-nucleoside agonists at the A_2B_ adenosine receptor. In Silico Pharmacol..

[35] Dal Ben D., Buccioni M., Lambertucci C., Marucci G., Santinelli C., Spinaci A., Thomas A., Volpini R. (2016). Simulation and Comparative Analysis of Different Binding Modes of Non-nucleoside Agonists at the A_2A_ Adenosine Receptor. Mol. Inform..

[36] Rodriguez D., Gao Z.G., Moss S.M., Jacobson K.A., Carlsson J. (2015). Molecular docking screening using agonist-bound GPCR structures: Probing the A_2A_ adenosine receptor. J. Chem. Inf. Model..

[37] Xu F., Wu H., Katritch V., Han G.W., Jacobson K.A., Gao Z.G., Cherezov V., Stevens R.C. (2011). Structure of an agonist-bound human A_2A_ adenosine receptor. Science.

[38] Lebon G., Bennett K., Jazayeri A., Tate C.G. (2011). Thermostabilisation of an agonist-bound conformation of the human adenosine A_2A_ receptor. J. Mol. Biol..

[39] Pires D.E., Blundell T.L., Ascher D.B. (2015). pkCSM: Predicting small-molecule pharmacokinetic and toxicity properties using graph-based signatures. J. Med. Chem..

[40] Lipinski C.A., Lombardo F., Dominy B.W., Feeney P.J. (2001). Experimental and computational approaches to estimate solubility and permeability in drug discovery and development settings. Adv. Drug Deliv. Rev..

[41] Pereira A.S.P., den Haan H., Peña-García J., Moreno M.M., Pérez-Sánchez H., Apostolides Z. (2019). Exploring african medicinal plants for potential anti-diabetic compounds with the DIA-DB inverse virtual screening web server. Molecules.

[42] Vincenzi F., Targa M., Romagnoli R., Merighi S., Gessi S., Baraldi P.G., Borea P.A., Varani K. (2014). TRR469, a potent A_1_ adenosine receptor allosteric modulator, exhibits anti-nociceptive properties in acute and neuropathic pain models in mice. Neuropharmacology.

[43] Varani K., Massara A., Vincenzi F., Tosi A., Padovan M., Trotta F., Borea P.A. (2009). Normalization of A_2A_ and A_3_ adenosine receptor up-regulation in rheumatoid arthritis patients by treatment with anti-tumor necrosis factor alpha but not methotrexate. Arthritis Rheum..

[44] Varani K., Merighi S., Gessi S., Klotz K.-N., Leung E., Baraldi P.G., Cacciari B., Romagnoli R., Spalluto G., Borea P.A. (2000). [^3^H]MRE 3008F20: A novel antagonist radioligand for the pharmacological and biochemical characterization of human A_3_ adenosine receptors. Mol. Pharmacol..

[45] Ravani A., Vincenzi F., Bortoluzzi A., Padovan M., Pasquini S., Gessi S., Merighi S., Borea P.A., Govoni M., Varani K. (2017). Role and function of A_2A_ and A_3_ adenosine receptors in patients with ankylosing spondylitis, psoriatic arthritis and rheumatoid arthritis. Int. J. Mol. Sci..

