# Supplementary material for: Amino-3,5-Dicyanopyridines Targeting the Adenosine Receptors. Ranging from Pan Ligands to Combined A1/A2B Partial Agonists
Source: Pharmaceuticals (Basel). 2019 Oct 22;12(4):159. doi: 10.3390/ph12040159 (PMC6958422; doi:10.3390/ph12040159)
Supplement: Supplementary file 1 [file pharmaceuticals-12-00159-s001.pdf]

Amino-3,5-dicyanopyridines targeting the adenosine receptors. Ranging from pan ligands to combined A_1/A_{2B} partial agonists

Daniela Catarzi,[a] Flavia Varano,[a] Katia Varani,[b] Fabrizio Vincenzi,[b] Silvia Pasquini,[b] Diego Dal Ben,[c] Rosaria Volpini,[c] Vittoria Colotta[a]*

Table S1. Combustion Analysis data of the newly synthesized compounds **1-4**, **6-20**, **26** and **37**, and derivative **33** [20].

compd	Formula	C	H	N
		**calcd-found**	**calcd-found**	**calcd-found**
**1**	$C_{22}H_{20}N_6OS$	63.44-63.25	4.84-4.92	20.18-20.04
**2**	$C_{21}H_{20}N_6OS$	62.36-62.56	4.98-4.75	20.78-20.96
**3**	$C_{20}H_{16}N_6OS$	61.84-61.98	4.15-3.98	21.63-21.84
**4**	$C_{21}H_{18}N_6OS$	62.67-62.92	4.51-4.34	20.88-20.67
**6**	$C_{19}H_{15}N_7OS$	58.60-58.35	3.88-4.03	25.18-25.36
**7**	$C_{17}H_{11}ClN_6S$	55.66-55.39	3.02-3.25	22.91-22.75
**8**	$C_{17}H_{11}FN_6S$	58.28-58.36	3.16-3.35	23.99-23.75
**9**	$C_{18}H_{11}F_3N_6S$	54.00-53.82	2.77-2.56	20.99-20.82
**10**	$C_{18}H_{14}N_6S$	62.41-62.78	4.07-4.24	24.26-24.42
**11**	$C_{18}H_{14}N_6S_2$	57.12-57.35	3.73-3.59	22.21-22.34
**12**	$C_{18}H_{14}N_6S$	62.41-62.26	4.07-4.32	24.26-24.02
**13**	$C_{12}H_{10}N_6S$	53.62-53.45	3.73-3.92	31.09-30.86
**14**	$C_{17}H_{13}N_7$	64.75-64.93	4.16-4.34	31.09-30.85
**15**	$C_{15}H_{13}N_5O$	64.51-64.34	4.69-4.81	25.07-24.89
**16**	$C_{22}H_{19}N_5O$	71.53-71.38	5.18-5.34	18.96-18.74
**17**	$C_{16}H_{10}N_6$	67.12-67.34	3.52-3.28	29.35-29.16
**18**	$C_{13}H_{18}N_4$	70.90-70.72	3.66-3.52	25.44-25.23
**19**	$C_{16}H_{15}N_5O_2$	62.13-62.01	4.89-5.05	22.64-22.41
**20**	$C_{17}H_{14}N_4O_3S$	57.62-57.86	3.98-3.72	15.81-16.03
**26**	$C_{20}H_{14}N_4S$	70.15-69.08	4.12-4.34	16.36-16.18
**33**	$C_{13}H_7ClN_4S$	54.45-54.31	2.46-2.53	19.54-19.67
**37**	$C_{14}H_{10}N_4S_2$	56.35-56.06	3.38-3.54	18.78-18.96
**38**	$C_{20}H_{14}N_4S$	63.14-63.35	3.78-3.65	21.04-20.94

Table S2. Calculated[a] molecular properties and ADMET parameters for compound **3**.

**Molecular properties**			
**Descriptor**	**Predicted value**		
Molecular weight	388.456		
LogP	3.65436		
# Rotatable bonds	7		
# H-bond acceptors	7		
# H-bond donors	2		
Surface Area	166.056		
**ADMET parameters**			
**Property**	**Model name**	**Predicted value**	**Unit**
Absorption	*Water solubility*	-3.049	log mol/L
Absorption	*Caco2 permeability*	0.183[c]	log Papp in 10-6 cm/s
Absorption	*Intestinal sbsorption (h)*[b]	86.892[d]	% Absorbed
Toxicity	*AMES toxicity*	YES	YES/NO
Toxicity	*Max. tolerated dose (h)*	0.501[e]	log mg/kg/day
Toxicity	*hERG I inhibitor*	NO	YES/NO
Toxicity	*hERG II inhibitor*	YES	YES/NO
Toxicity	*Oral rat acute toxicity (LD50)*	2.559	mol/kg
Toxicity	*Hepatotoxicity*	YES	YES/NO

[a] http://biosig.unimelb.edu.au/pkcsm/;
[b]human;
[c]high Caco2 permeability > 0.90;
[d]Intestinal sbsorption (h) ≥ 30%;
[e]Max. tolerated dose (h): low when ≤ 0.477; high when ≥ 0.477.
